# A comparison of the effectiveness of three parenting programmes in improving parenting skills, parent mental-well being and children's behaviour when implemented on a large scale in community settings in 18 English local authorities: the parenting early intervention pathfinder (PEIP)

**DOI:** 10.1186/1471-2458-11-962

**Published:** 2011-12-30

**Authors:** Geoff Lindsay, Steve Strand, Hilton Davis

**Affiliations:** 1 Centre for Educational Development, Appraisal and Research (CEDAR), University of Warwick, Kirby Corner Road, Coventry CV4 7AL, UK; 2 King's College London/Institute of Psychiatry, Centre for Parent and Child Support, 66 Snowsfield, Guy's Hospital, London SE1 3SS, UK; 3 Brook Cottage, Colway Lane, Lyme Regis, Dorset DT7 3BG, UK

## Abstract

**Background:**

There is growing evidence that parenting programmes can improve parenting skills and thereby the behaviour of children exhibiting or at risk of developing antisocial behaviour. Given the high prevalence of childhood behaviour problems the task is to develop large scale application of effective programmes. The aim of this study was to evaluate the UK government funded implementation of the Parenting Early Intervention Pathfinder (PEIP). This involved the large scale rolling out of three programmes to parents of children 8-13 years in 18 local authorities (LAs) over a 2 year period.

**Methods:**

The UK government's Department for Education allocated each programme (Incredible Years, Triple P and Strengthening Families Strengthening Communities) to six LAs which then developed systems to intervene using parenting groups. Implementation fidelity was supported by the training of group facilitators by staff of the appropriate parenting programme supplemented by supervision. Parents completed measures of parenting style, efficacy, satisfaction, and mental well-being, and also child behaviour.

**Results:**

A total of 1121 parents completed pre- and post-course measures. There were significant improvements on all measures for each programme; effect sizes (Cohen's *d*) ranged across the programmes from 0.57 to 0.93 for parenting style; 0.33 to 0.77 for parenting satisfaction and self-efficacy; and from 0.49 to 0.88 for parental mental well-being. Effectiveness varied between programmes: Strengthening Families Strengthening Communities was significantly less effective than both the other two programmes in improving parental efficacy, satisfaction and mental well-being. Improvements in child behaviour were found for all programmes: effect sizes for reduction in conduct problems ranged from -0.44 to -0.71 across programmes, with Strengthening Families Strengthening Communities again having significantly lower reductions than Incredible Years.

**Conclusions:**

Evidence-based parenting programmes can be implemented successfully on a large scale in community settings despite the lack of concentrated and sustained support available during a controlled trial.

## Background

Behavioural, emotional and social difficulties (BESD) are common among children and young people. Within the UK studies have found prevalence rates of 10% for 5-16 year olds having a clinically diagnosed mental disorder, including 6% with a conduct disorder [1] and one fifth of parents of 2-8 year olds reporting difficulties with their child's behaviour [2]. Conduct problems in early and middle childhood are associated with increased risks during late adolescence and early adulthood of crime, mental health problems, relationships and parenthood difficulties, and substance dependence [3,4]; indeed risks persist until 29-33 years [5].

As parents are fundamental to their children's development, there has been considerable interest in the development of direct training to enhance parental understanding and skills in order to prevent the development of behavioural difficulties [6]. Delivery by parenting group provides an approach that may be more cost-effective than individual interventions and also provides the opportunity for mutual peer support. Universal prevention programmes address a number of limitations posed by targeted provision, including stigmatization of parents by their attendance; non-delivery of service to those misclassified by the selection criteria; and delivery to the highest risk groups only, whereas the majority of children with later mental health problems come from the larger lower risk population [7].

Systematic reviews have provided evidence of the *efficacy *of group-based parenting programmes for improving parenting, parental mental health and the social and emotional development of their children [8-12]. For example, a review by Brestan and Eyberg [13] of 82 studies of treatment for children with conduct problems identified 20 studies reporting treatments they described as 'probably efficacious' and two interventions that met their strongest criteria for well-established treatment, one of which was Webster-Stratton's programme for parents of 4-8 year olds with behavioural problems, which became the Incredible Years programme. A meta-analysis of 55 evaluations of Triple-P by Nowak and Heinrichs [14] found positive effects for both parenting and child problem measures, with effect sizes ranging between 0.35 and 0.48 for between groups post-intervention comparisons; analysis of follow up scores indicated that intervention effects were maintained, although they did not improve further.

Well conducted trials of parenting programmes are typically small scale: the mean number of parents for 27 of the 28 randomized control trials (RCTs) reviewed by Nowak and Heinrichs [14] was 86 (range 21-305) prior to randomization. Consequently, the mean number in the treatment groups is likely to have been about 40-50. A systematic review of 57 RCTs by Dretzke *et al. *[10] reports a mean group size of just 21 parents.

In order to be considered suitable for large scale implementation, it is necessary not only to demonstrate a programme's *efficacy *under well controlled and supported trial conditions but also its *effectiveness *when implemented on a large scale under real-world conditions (Society for Prevention Research [15]). Scaling up from an efficacy to an effectiveness study raises new challenges. There are likely to be differences in the control of fidelity to the programme that is possible in the efficacy trials compared with that possible with real world implementation. Manualised guidance with rigorous systematic training addresses this issue but there remain other challenges including those related to the leadership, commitment and support available within real world settings: levels of commitment and support are likely to be lower and quality of leadership may be more variable. It is necessary that the intervention is consistent with the aims and needs of the host organisation if implementation and its evaluation are to be practical.

Participant selection is likely to be more problematic for real world effectiveness research in community settings as the host organisation will have its own policy for providing services. Efficacy trials of parenting programmes typically select parents against pre-determined criteria, which may lead to the exclusion of parents who might benefit from participation in the programme. Such a loss of potential participants can be unacceptable to a community service.

Amount of evidence for efficacy varies between programmes. Comparative studies of different programmes are not common but evidence for the outcomes from different parenting programmes is important to guide policy for large scale implementation [16].

This paper reports the evaluation of a large scale implementation of three parenting programmes, which have evidence of efficacy, in community settings in England: the Parenting Early Intervention Pathfinder (PEIP). This was an initiative funded by the UK government's Department for Children, Schools and Families (DCSF), now the Department for Education (DfE), at £7.6 million over 2 years in 18 local authorities (LAs) in England. Part of the Respect Action Plan [17] intended to prevent crime, tackle antisocial behaviour and enhance communities, the Pathfinder was designed to support parents of young people aged 8-13 years demonstrating or at risk of developing behavioural difficulties by the funding of three parenting programmes delivered in community settings. This age group was selected by the government as this period was considered to be under-resourced. It is also under-researched as evidence for the effectiveness of parenting programmes has typically been based on parents of younger children.

### Aims

The study had two aims.

1. To examine whether the effectiveness of three evidence based parenting programmes was maintained when implemented on a large scale in community settings.

2. To compare the effectiveness of the three parenting programmes, as perceived by parents, in improving: a) parenting skills, b) parental mental well-being, and c) behaviour in children exhibiting or at risk of conduct problems.

## Methods

### Design

The Department for Children, Schools and Families (DCSF) selected three manualised parenting programmes on the basis of a review of their efficacy [11] and selected 18 local authorities (LAs) in England with prior experience of parenting support to receive funding to implement one of the three programmes over the period 2006-08 (6 LAs per programme). Allocation of programmes to the LAs was conducted by the DCSF prior to the engagement of the research team. It was not possible to include a control group in the design as all funding to LAs was intended for implementation of their specified programme and the LAs had no waiting list system. However, there was random allocation to the LAs of the three parenting programmes which all had prior evidence of efficacy. In addition, this design allowed comparison of the effectiveness of their implementation across 18 LAs.

Although the DCSF funded the PEIP as part of government policy, LAs had a great deal of autonomy in its implementation. The DCSF specified recruitment criteria, namely that parents had a child aged between 8 and 13 years who was engaging in or at risk of developing antisocial behaviour. However, no systematic audit was carried out by the DCSF to hold the LAs to account and no formal guidance on implementation was provided. Limited support was provided by a one-day start-up conference when lead officers from the LAs were briefed on government policy on parenting support by DCSF officials. The evaluation was explained by the research team and representatives from the three programmes briefed their respective LAs and set up networks to organise training and support. Implementation was then determined at LA level. Each LA developed its own infrastructure to support the PEIP, including management/coordination, access to training for facilitators by the parenting programmes' staff, and delivery of parenting courses.

### The programmes

#### Incredible years

The Incredible Years programme [18] was developed in the USA to be delivered to groups of parents of children aged 0-8 years. The focus of the programme is the enhancement of effective, positive parenting, so as to enable children's development and education and to manage behavioural problems where necessary. However, there is also a strong concern with parents' adaptation more generally so that they are better able to deal with their own problems and relationships. There is substantial evidence for its efficacy in improving parenting skills and reducing child conduct problems from randomized trials (e.g. [13,19,20]). There is also evidence of long-term maintenance of gains over 10 years [21].

#### Triple P

The Triple P Positive Parenting Program [22] was developed in Australia. It differs from other parenting programmes in comprising a complex system of interventions grouped into five levels, reflecting increasing complexity and severity of need. The levels range from community information provision to intensive one-to-one work. The evaluation within this study was concerned with the implementation of courses for groups of parents with the focus on parental management of child behaviour and reduction of parental stress. Core principles in the courses included enabling parents to provide a safe and interesting environment for their children, a positive learning environment and assertive discipline, while maintaining realistic expectations and taking care of themselves as parents. The effectiveness of Triple P has been demonstrated for different variants including Level 4, which was the main intervention used in the PEIP. A meta-analysis of 55 studies [14] concluded that Triple P causes positive changes in the small to medium range for child problem behaviour, parent well-being and parenting skills; effect sizes increased with the intensity level of the programme with overall effect sizes (Cohen's *d*) ranging between 0.35 and 0.48 for between groups comparisons.

#### Strengthening families strengthening communities

The third programme, Strengthening Families Strengthening Communities (SFSC), was developed in the USA, predominantly for minority ethnic groups [23]. As with the other programmes it was implemented in a group format with the central concern being the development of effective parenting skills. However, there are broader themes involved in the course which include cultural and spiritual dimensions, enhancing relationships, rites of passage and community involvement. There is evidence of efficacy from pre- to post-assessment studies including one in the UK [24,25] but no evidence is yet available from a randomised control trial.

All the programmes are carefully manualised with elaborate procedures for training and supervision (see [26] for more details). For the PEIP project, the Incredible Years used their basic and advanced programmes adapted to cover the age range 8-13 years. This usually involved 17 two-hour sessions (34 h). The other programmes were implemented as normal: Triple P Level 4 comprised five two-hour face-to-face sessions and three on the telephone (11.5 h) and SFSC 12 three-hour sessions (36 h). The 266 groups (56 Incredible Years, 142 Triple P and 68 SFSC) typically ran weekly except for holiday periods, and were accommodated in community settings including schools. The three programmes differed in length, content and structure but were all group based with a prime focus on developing parenting knowledge and skills.

### Participants

The parents (*N *= 2207: 86.7% female, 13.3% male) were recruited locally by each of the 18 LAs. Parents were asked to select the child whose behaviour gave them most concern to be their target child for the purpose of assessment. Table 1 presents the demographic characteristics of the parents and their children at baseline for each programme and the total sample. The majority of parents (91.2%) were biological parents, predominantly White British (76.1%) but with a substantial minority (11.9%) of South East Asian heritage, primarily Pakistani (2.6%) and Bangladeshi (7.3%). Most were socio-economically disadvantaged: 46.7% had left school at 16 years or earlier (the end of compulsory education in England) and 52.3% had a weekly income of no more than £200. The majority of target children, mean age 9.2 years (*SD *= 3.2), were boys (62.3%: 37.7%). A significantly higher proportion of Incredible Years parents left school at age 16 or earlier, and a lower percentage had attended university (χ^2 ^= 57.4, *df *= 8, *p <*.001). A significantly greater proportion of parents taking Triple P were in the highest two income bands (χ^2 ^= 60.3, *df *= 10, *p *< .001). There was a significantly higher percentage of parents from minority ethnic groups, particularly Indian, Pakistani & Bangladeshi, attending SFSC (χ^2 ^= 559, *df *= 12, *p *< .001). Lastly, the mean age of Incredible Years children was significantly lower (*F *= 35.0, *df *= 2, 1969, *p *< .001).

**Table 1:** Descriptive statistics for parental and pupil background measures by parenting programme

		Parenting Programme						Total	
				
		SFSC		Incredible Years		Triple P			
		
Variable	Value	N	%	N	%	N	%	N	%
Parent gender	Male	79	12.2	55	11.7	157	14.6	291	13.3
(n = 2194)	Female	567	87.8	415	88.3	921	85.4	1903	86.7

Relationship to the child	Biological parent	552	92.9	411	91.5	953	90.0	1916	91.2
(n = 2102)	Step parent	10	1.7	14	3.1	30	2.8	54	2.6
	Parent's partner	16	2.7	3	0.7	20	1.9	39	1.9
	Adoptive parent	2	0.3	2	0.4	8	0.8	12	0.6
	Foster parent	6	1.0	3	0.7	21	2.0	30	1.4
	Other	8	1.3	16	3.6	27	2.5	51	2.4

Parent ethnic group*	White British	275	46.8	413	89.4	783	88.7	1471	76.1
(n = 1932)	White other groups	23	3.9	15	3.2	34	3.9	72	3.7
	Mixed heritage	20	3.4	11	2.4	19	2.2	50	2.6
	Asian	199	33.9	13	2.8	17	1.9	229	11.9
	Black	46	7.8	7	1.5	16	1.8	69	3.6
	Any other ethnic group	24	4.1	3	0.6	14	1.6	41	2.1

Parent highest level of education*	Left school at 16 or earlier	265	45.7	260	60.2	416	41.8	941	46.9
(n = 2008)	Left school at 17 or 18	82	14.1	39	9.0	99	9.9	220	11.0
	FE college/apprenticeship	161	27.8	98	22.7	321	32.2	580	28.9
	Attended university	72	12.4	35	8.1	160	16.1	267	13.3

Parent weekly income*	£150 or less	174	36.9	154	35.2	275	30.4	603	33.2
(n = 1814)	£150-£200	115	24.4	95	21.7	136	15.0	346	19.1
	£201-£250	46	9.7	63	14.4	113	12.5	222	12.2
	£251-£350	78	16.5	65	14.8	162	17.9	305	16.8
	£351 or above	59	12.5	61	13.9	218	24.1	338	18.6

Child gender	Male	367	65.0	270	63.5	610	60.3	1247	62.3
(n = 2002)	Female	198	35.0	155	36.5	402	39.7	755	37.7

Child age (n = 1972)*	Mean (*SD*)	9.7	(3.0)	8.1	(3.0)	9.4	(3.2)	9.2	(3.2)

Completing post-test									
		56.3%		50.7%		47.5%		50.8%	

Total sample		650		473		1084		2207	

*Notes: *1. *SFSC *Strengthening Families Strengthening Communities

2. Percentages are based on valid values only

3. * indicates a significant difference (*p* < .001 in all cases) between programmes using chi-squared tests and *Z *tests for comparing column proportions (except child age which was tested by one-way ANOVA)

### Intervention

Implementation was representative of the way these three programmes are typically conducted. The three programmes described above were delivered to groups of parents typically comprising about 10 parents. The sessions were conducted by people (referred to as facilitators) specially trained for the purpose. Group sessions were guided by the programme manual to optimise fidelity and comprised watching programme videos (DVDs) created as stimulus material for specific teaching points, group discussions and role play. Parents had their own personal handbook and carried out homework between sessions.

Facilitators (total 1100), from a range of backgrounds including social workers, psychologists and health visitors, were recruited by each LA and trained by the relevant programme provider over 3-5 days, according to the programme's usual training requirements. Supervision and follow-up checks on implementation fidelity were made subsequent to initial training in accord with each programme's normal practice. This included observation by experienced staff from the programmes as well as local senior facilitators. The parenting programmes were delivered in a variety of settings including community centres, schools, clinics, and the premises of voluntary bodies: this range is representative of the locations typically used for these programmes.

### Outcome measures

The effectiveness of the parenting programmes was measured by the following primary outcome measures; there were no secondary outcomes to be measured. The instruments were selected to measure the domains expected to show improvements following attendance at the programme and common to all three programmes.

#### Parent mental well-being

The Warwick-Edinburgh Mental Well-being Scale (WEMWBS) [27] has 14 items rated on 5-point scales. It has been standardised on a UK population and measures positive mental health, including subjective experience of happiness and life satisfaction, and perspectives on psychological functioning and personal relationships. Tennant *et al. *report that the WEMWBS has moderate to high levels of construct validity with nine other comparable scales (median .73, range .42-.77). Internal consistency in the present study was high (Cronbach's alpha .93). The national median is 51 (inter-quartile range 45-56).

#### Parenting style

The Parenting Scale-Adolescent [28] is a shortened form of a 30 item scale of the same name originally developed for parents of pre-school children [29]. It is a widely used measure of parenting styles comprising 13 items, each scored on a 7-point scale: two sub-scales, Laxness and Over-reactivity, together with a single item, Monitoring, are aggregated to produce a Total Score. Internal consistency was high, Cronbach's alpha: .82 Laxness, .83 Over-reactivity and .84 Total Score. While national norms are not available for the adolescent version it is more appropriate for the children in the age range included in this study.

Being a Parent is an adaptation of the Parenting Sense of Competence Scale (PSOC) [30]. The 16 items measured on 6-point scales form two sub-scales following Johnston and Mash [31], whose two factor solution improved on the original 17 item PSOC. Parenting Satisfaction (9 items) is an affective dimension reflecting parental frustration, anxiety and motivation and Parenting Efficacy (7 items) an instrumental dimension reflecting perceived competence, problem-solving ability and capability in the parental role. The two scales are aggregated to produce a Total Score. Internal consistency was satisfactory, with alphas of .75 for Satisfaction .76 Efficacy and .79 Total Score. Normative data from a random sample of 297 Canadian mothers of children aged 4-9 are presented in Johnston & Mash [31].

Evidence for validity is well established through many studies which have used these scales, in the form of positive correlations with direct observation of parenting behaviour (e.g. [28,32]),

#### Child behaviour

Behaviour problems of the child about whom the parent was most concerned were measured using the Strengths and Difficulties Questionnaire (SDQ) [33]. The 25 items are scored on 3 point scales (not true = 0, somewhat true = 1, certainly true = 2). These produce four 'problem' scales, Emotional Symptoms, Conduct Problems, Hyperactivity, and Peer Problems, each with five items (range 0-10) which sum to a Total Difficulties score (range 0-40). A five item Prosocial scale (range 0-10) measures positive behaviours and an Impact score is also produced. Raw scores and clinical categorical scores (normal, borderline and abnormal) based on a representative sample of 10,298 British children aged 5-15 are available [34]. Internal consistency was satisfactory with alphas of .71 Conduct Problems, .65 Impact and .83 Total Difficulties. There is extensive evidence for the validity of the SDQ as a measure of children's behaviour [35].

### Procedure

Parents completed the questionnaires, which were then returned to the research team for analysis, during or just prior to the first session (pre-course) and again during the last session of their parenting programme (post-course).

### Statistical analyses

Intention to treat (ITT) has become the preferred method of analysis for intervention trials. This analysis includes all participants in the groups to which they were randomly assigned, regardless of the intervention received, or their continuation or withdrawal from the intervention or any deviations from the trial protocol. Interpretation of an ITT analysis is problematic when there is a substantial loss of participants after the pre-intervention measures. Imputation of outcome scores may be made, for example by using the last observation carried forward, but this is likely to attenuate any effects [36]. The greater the loss of outcome data the more conservative will be the estimate of effects.

An ITT analysis is therefore particularly problematic for studies of effectiveness interventions in community 'real life' settings where higher attrition is likely compared with a small scale trial. In the present study pre-course data were available on 2207 parents and post course data were received from 1121 or 50.8% of parents: 240 (50.7%) Incredible Years, 515 (47.5%) Triple P and 366 (56.3%) SFSC. The level of post-course response was a result not only of true attrition (participants dropping out of their parenting groups) but also of administrative errors by LA staff, resulting in questionnaires not being returned to the research team, estimated at around 20% of parents.^1 ^As argued above, imputing outcome scores where around half the sample were missing would be inappropriate. A per protocol (PP) analysis was therefore conducted on data from all parents who completed both pre- and post-course measures.

In undertaking the PP analysis it is important to determine whether the parents who responded at post-test differed significantly from those who did not respond in terms of demographics and pre-course measures. With respect to demographics there were significant differences only for income and education: parents responding to both pre- and post-course questionnaires were more likely to be drawn from the higher income groups (e.g. 42% vs. 37% had weekly income of £200 or above, *χ*^2 ^= 17.3, *df *= 5, *p *= .008) and had a higher level of education (e.g. 44% vs. 50% left school at 16 or earlier, *χ*^2 ^= 13.6, *df *= 4, *p *= .009). However with respect to the key parenting and child measures, there was a statistically significant difference on only one of the 14 measures. Those completing the post-course measures initially reported slightly lower scores for child Conduct Problems (*Mean (SD*) = 4.3 (2.4) vs. 4.6 (2.6), *p *= .009) compared to those who did not respond post-course, although the difference in means represents only 0.12 of a *SD*. Given there were no significant differences on 13 of the 14 measures, and the statistically significant difference that did exist was extremely small, there is little evidence to suggest those responding post-course differed substantially from those not responding on key parenting and child measures. We therefore consider it reasonable to conduct an analysis of change for those parents providing both pre- and post-course data.

Change scores (defined as post-course score minus pre-course score) were calculated for all scales. Analysis of variance (ANOVA) of change from baseline was employed to assess the effect of programme on improvement while controlling for demographic variables. ANOVA of change scores was employed rather than analysis of covariance (ANCOVA) with pre-course score as a covariate. This was because a review of the literature indicated that, while ANCOVA may have greater power in randomised designs, ANOVA of change scores is less biased in non-randomised studies of pre-existing groups [37] because the assumption in ANCOVA of equal pre-test means is often violated. Indeed in the present study there were significant differences between programmes in pre-course mean scores.

In the following sections separate results are presented for parent and child outcomes. Effect sizes (Cohen's *d*) are reported for each programme.^2 ^Programme comparisons are adjusted through ANOVA to control for differences between programmes in parent gender, education and income and child gender and age. In the absence of a suitable control group Triple P was used as the reference group as it was the largest, accounting for 47% of all post-course respondents. Bonferroni post hoc tests are reported where significant (alpha level of .05 for all statistical tests).

### Ethical approval

Ethical approval was given by the University of Warwick Humanities and Social Sciences Research Ethics Committee (Ref: Eth. App. 12/06-07). All participants were given information about the study and gave informed consent for their involvement. They were also informed that all data would be anonymised, they were free to withdraw at any time and have their data removed.

## Results

### Parental mental well-being

**Figure 1:**
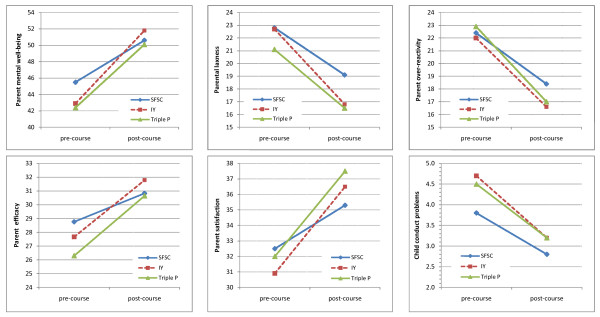
**Pre and post-course means for each programme for: (a) parent mental well-being; (b) parent laxness; (c) parent over-reactivity, (d) parent efficacy; (e) parent satisfaction; and (f) child conduct problems**.

As parents started their parenting course their mental well-being was low: SFSC median = 42.3, Incredible Years median = 46.1 and Triple P median = 41.8 compared with the national median of 51 and national 25th centile score of 43. Mental well-being scores increased for each programme to the national median or above by the end of their courses, but parents completing the SFSC showed significantly less improvement than both Incredible Years (*p *< .001) and Triple P (*p *= .01), see Table 2 and Figure 1a).

**Table 2:** Parenting behaviours: Effect size by programme and multiple comparisons between programmes

	SFSC				Incredible Years				Triple P				ANOVA	ANOVA multiple comparisons Mean difference (CIs)			
**Variable**	**N**	**Before** **(Mean, SD)**	**After** **(Mean, SD)**	**Effect size**	**N**	**Before** **(Mean, SD)**	**After** **(Mean, SD)**	**Effect size**	**N**	**Before** **(Mean, SD)**	**After** **(Mean, SD)**	**Effect size**	**F-test (*df*)** ***p-*value**	**SFSC-IY**	**SFSC-TP**	**IY-TP**	**Summary**

Mental Well-Being	347	45.3 (11.2)	50.6 (10.3)	**0.49**	237	42.9 (10.3)	51.8 (10.0)	**0.88**	487	42.4 (9.7)	50.1 (9.3)	**0.81**	*F*(2,962) = 8.75, *p *= .001	-3.65 *** (-5.70, -1.60)	-2.11 ** (-3.81, -0.40)	1.54 (-0.36, 3.44)	SFSC < (IY = TP)

Parental laxness	325	22.8 (6.7)	19.1 (6.2)	**-0.57**	229	22.7 (7.1)	16.8 (6.1)	**-0.89**	486	21.1 (6.1)	16.5 (6.2)	**-0.74**	*F*(2,938) = 3.88, *p *= .021	2.01 ** (0.57, 3.49)	0.73 (-0.48, 1.95)	-1.29 (-2.63, 0.43)	SFSC < IY

Parent over-reactivity	314	22.4 (6.0)	18.4 (6.1)	**-0.65**	230	22.0 (6.4)	16.6 (6.2)	**-0.86**	488	22.9 (6.5)	17.0 (6.1)	**-0.93**	*F*(2,930) = 5.36, *p *= .005	1.06 (-0.46, 0.56)	1.83 ** (0.56, 3.11)	0.77 (-0.62, 2.16)	SFSC < TP

Parenting Efficacy	329	28.8 (6.6)	30.8 (5.9)	**0.33**	236	27.7 (6.8)	31.8 (6.1)	**0.64**	481	26.3 (5.9)	30.6 (5.4)	**0.77**	*F*(2,943) = 8.03, *p *= .001	-1.91 ** (-3.26, -0.57)	-2.02 *** (-3.15, -0.89)	-0.11 (-1.35, 1.13)	SFSC < (IY = TP)

Parenting Satisfaction	329	32.5 (8.1)	35.3 (8.5)	**0.34**	235	30.9 (7.7)	36.5 (7.9)	**0.72**	484	32.0 (7.4)	37.5 (7.4)	**0.74**	*F*(2,946) = 11.78, *p *= .001	-3.14 *** (-4.76, -1.52)	-2.67 *** (-4.01, -1.32)	0.46 (-1.04, 1.95)	SFSC < (IY = TP)

*Notes: *1. Effect sizes are Cohen's *d*

2. Programme comparisons based on ANOVA of the change from baseline, defined as post-test minus pre-test, with parent gender, education and income and child gender and age included as control variables.

3. Multiple comparisons completed using Bonferroni test: n.s. = not significant;*p < .05; **p < .01; ***p < .001

### Parenting behaviour

Preliminary analyses revealed that the correlations between the pre-course parenting measures were small to modest (highest -.40 between Satisfaction and Over-reactivity) indicating that Laxness, Over-reactivity, Parenting Efficacy and Satisfaction with being a Parent were measuring largely independent aspects of parenting.

Normative data were not available for Laxness and Over-reactivity, but parents started their parenting course with mean Parenting Satisfaction scores (Table 2) around 1 *SD *below the normative sample (*M *= 38.1, *SD *= 6.2) although their Parenting Efficacy scores were slightly above the norm (*M *= 25.2, *SD *= 5.9). Parents from all three programmes showed improvement on each of the parenting measures, with the effect size for reductions in laxness and over-reactivity ranging from -0.57 to -0.93, and improvements in parenting efficacy and satisfaction with being a parent ranging from 0.33 to 0.74 (Table 2). These results indicate that after attending any one of these parenting programmes parents were less likely to give way inappropriately to their child; less likely to overreact when their child misbehaved; more likely to deal with the child calmly; to feel more effective as a parent; and to have an improved sense of satisfaction with being a parent.

However improvements varied across programmes with SFSC the least effective programme on all measures (Table 2; see also Figure 1b-e). For Laxness the effect size for SFSC (-0.57) was substantially lower than for Incredible Years (-0.89), *p *= .003, and for Over-reactivity the effect size for SFSC (-0.65) was substantially lower than for Triple P (-0.93), *p *= .002. For Parenting Efficacy the effect size for SFSC (0.33) was around half the magnitude of both Incredible Years (0.64) and Triple P (0.77), *p *= .002 and *p *< .001 respectively; for Parenting Satisfaction, again the effect size for SFSC (0.34) was around half the magnitude of both Incredible Years (0.72) and Triple P (0.74), both *p *< .001.

### Child behaviour

At the start of the programmes high levels of child conduct problems were reported by parents with the mean pre-course Conduct Problems scores ranging from 3.8 to 4.7 (Table 3), around 0.9-1.4 *SD *above the UK average (*M *= 1.9, *SD *= 2.0). For SDQ Total Difficulties the mean pre-course scores ranged from 16.4 to 18.4 (Table 3), around 1.4-1.7 *SD *above the UK average (*M *= 8.4, *SD *= 5.8). In terms of the proportion of children in the 'abnormal' category the figures for Conduct Problems were: SFSC 52.9%; Incredible Years 69.0%; and Triple P 64.6%, compared with the norm of 12.7%; and for SDQ Total Difficulties: SFSC 53.9%, Incredible Years 60.4%, and Triple P 58.6%, compared with the norm of 9.8%.

**Table 3:** Child behaviours: Effect size by programme and multiple comparisons between programmes

	SFSC				Incredible Years				Triple P				ANOVA	ANOVA multiple comparisons Mean difference (CIs)			
**SDQ Variable**	**N**	**Before** **(Mean, SD)**	**After** **(Mean, SD)**	**Effect size**	**N**	**Before** **(Mean, SD)**	**After** **(Mean, SD)**	**Effect size**	**N**	**Before** **(Mean, SD)**	**After** **(Mean, SD)**	**Effect size**	**F-test (*df*)** ***p-*value**	**SFSC-IY**	**SFSC-TP**	**IY-TP**	**Summary**

Emotional symptoms	341	3.6 (2.5)	2.6 (2.1)	**-0.43**	232	3.9 (2.3)	3.1 (2.5)	**-0.34**	494	3.8 (2.6)	2.7 (2.4)	**-0.45**	*F*(2,973) = 1.32 *p *= .269	n.s.	n.s.	n.s.	

Conduct problems	344	3.8 (2.4)	2.8 (2.1)	**-0.44**	232	4.7 (2.2)	3.2 (2.1)	**-0.71**	495	4.5 (2.4)	3.2 (2.2)	**-0.56**	*F*(2,975) = 2.69, *p *= .068	0.51** (0.09, 0.94)	0.27 (-0.08, 0.62)	-0.24 (-0.64, 0.15)	SFSC < IY

Hyper-activity	329	5.8 (2.6)	4.6 (2.4)	**-0.46**	231	6.5 (2.7)	5.4 (2.7)	**-0.41**	493	6.3 (2.7)	5.1 (2.7)	**-0.43**	*F*(2,964) = 0.60, *p *= .548	n.s.	n.s.	n.s.	

Peer problems	337	3.3 (2.2)	2.9 (2.1)	**-0.19**	231	3.4 (2.2)	2.9 (2.2)	**-0.22**	496	3.2 (2.3)	2.6 (2.1)	**-0.28**	*F*(2,970) = 1.32, *p *= .268	n.s.	n.s.	n.s.	

Prosocial behaviours	341	6.8 (2.4)	7.1 (2.2)	**0.15**	232	6.5 (2.2)	7.3 (2.0)	**0.41**	495	6.2 (2.3)	6.8 (2.2)	**0.28**	*F*(2,972) = 4.12, *p *= .017	-0.55 ** (-0.99, -0.12)	-0.35 (-0.71, 0.02)	0.21 (-0.20, 0.61)	SFSC < IY

Impact	324	2.4 (2.5)	1.4 (2.3)	**- 0.38**	228	2.6 (2.4)	1.7 (2.4)	**- 0.38**	479	3.5 (2.9)	1.9 (2.4)	**-0.60**	*F*(2,937) = 5.61, *p *= .004	0.01 (-0.48, 0.49)	0.60 *** (-3.15, -0.89)	0.60 ** (0.15, 1.03)	(SFSC = IY) < TP

SDQ total difficulties	321	16.4 (6.8)	12.8 (6.6)	**- 0.55**	227	18.4 (6.7)	14.6 (7.0)	**-0.57**	490	17.8 (7.1)	13.6 (7.1)	**-0.59**	*F*(2,952) = 0.66, *p *= .518	n.s.	n.s.	n.s.	

*Notes: *See notes to Table 2

There were significant improvements in outcome scores for all programmes across all SDQ domains but particularly in Conduct Problems (effect sizes of -0.44, -0.71 and -0.56 for SFSC, Incredible Years and Triple P respectively) and SDQ Total Difficulties (-0.55, -0.57 and -0.59 respectively: see Table 3). These represent substantial reductions in the proportion of children in the 'abnormal' category for Conduct Problems to 33.1% for SFSC and 36.6% for Incredible Years and Triple P. Reductions were also found for Total Difficulties to 26.5% for SFSC, 40.1% for Incredible Years and 33.9% for Triple P. There were significant differences between programmes in their outcomes for conduct problems, prosocial behaviours and impact. Again SFSC was generally the least effective programme, with significantly lower improvements than Incredible Years for Conduct Problems (*p *= .012) and Prosocial behaviour (*p *= .007), and significantly lower reductions than Triple P for Impact of the difficulties (*p *= .001), for which Incredible Years was also significantly less effective than Triple P (*p *= .004).

## Discussion

This study examined the effectiveness of three evidence-based parenting programmes when rolled out on a large scale in community settings, as part of the Parenting Early Intervention Pathfinder (PEIP), and their relative effectiveness. The study found substantial improvements for all three programmes in parenting behaviour, parental mental well-being and reported behaviour of the child about whom the parent had most concern for displaying or being at risk of anti-social behaviour.

Initially the parents, most of whom were mothers, had low levels of mental well-being, and of both satisfaction and sense of efficacy as a parent. Their parenting style was characterised by high levels of impulsivity and over-reactivity. Prevalence of substantial behaviour problems was about six times the national average for their target child. Significant improvements were found for parenting skills and mental well-being following participation in one of the three programmes, with moderate to large effect sizes. Reported child behaviour also improved: conduct problems and SDQ total difficulties both reduced, although average effect sizes across programmes were lower than for the parenting measures. Other aspects of child behaviour, for example emotional symptoms and hyperactivity, showed less improvement, as expected, since these are not the main target of the programmes.

The significant improvements in both parents and children support previous studies. The magnitude of the effectiveness of the PEIP is encouraging given that this was a large scale roll out rather than a well controlled, smaller scale trial. For example, pre- to post-course improvements in child behaviour (SDQ conduct problems and total difficulties scores) for the Incredible Years sample are similar to those reported for a UK study of Incredible Years by Hutchings *et al. *[32]. The results for Triple P are comparable to those from a meta-analysis of 55 studies reported by Nowak and Heinrichs [14] for a within groups design (effect size range 0.45-0.57). Furthermore, the parents for whom we have post-course data are comparable on 13 of the 14 measures, indicating that the results are generalisable to the population from which the sample providing pre- to post-group comparison data was taken.

Comparison of the three programmes indicates that all were effective but there was a general trend for Strengthening Families Strengthening Communities (SFSC) to have lower effects than Incredible Years and Triple P. All three had been selected by the UK government as appropriate programmes to improve parenting skills and reduce children's behavioural difficulties; hence a three way comparison using measures selected to show improvements in the primary domains common to all three programmes is a reasonable analysis. However, in addition to these common aims, each programme had specific characteristics which were not examined in the study. It is not possible without more detailed research to determine with any certainty the reasons for SCSF being relatively less effective than the other two programmes. However, a possible explanation may be to do with the aims and content of SFSC being broader than Incredible Years and Triple P. For example, SFSC also emphasises concerns with the cultural, spiritual, ethnic and family issues related to child and family functioning and with the development of community involvement. The narrower focus of the other programmes on parenting and managing children's behaviour, with an emphasis on learning very specific practical skills in this area, may begin to explain the differences in outcomes.

The study was rigorous within the parameters of a large scale roll out of the programmes across 18 different local authorities (LAs). It comprised a large sample and appropriate measures of both parenting and child behaviour. There were, however, limitations. First, post-course data were available on only about half of the parents. This loss occurred partly because about a quarter of parents dropped out of their programme, a common phenomenon in parenting programmes especially when participants, as here, are subject to socioeconomic disadvantage and other adversities (e.g. Hutchings *et al. *[32] loss of 17%; Scott *et al. *[38,39] 19% in each study). However, at least a further 20% of the data was lost due to LA procedural errors, including failure to pass the measures on to, or collect them from, the parenting groups. While our analysis suggested few differences in pre-course scores between those who did or did not complete the post-course questionnaires, there were (small) differences in education and income demographics and the possibility of systematic bias cannot be eliminated.

Second, as a real world study, parents were not allocated randomly to the three programmes, possibly leading to bias. The government department funding the PEIP (Department for Children Schools and Families, now the Department for Education) selected 18 LAs judged to have more advanced practice in parenting support and allocated the LAs to programmes. Parents were recruited only to their LA's funded programme.

Third, unlike a trial, there was no information on the total population identified and the resulting drop out of potential parents before starting the parenting groups, for example refusal to participate. This can be substantial: from 240 to 153 (Hutchings *et al. *[32]) and 279 to 112 (Scott *et al. *[38]) in two recent UK studies. Local authorities recruited parents by various means, including referrals from other agencies and open advertisements. Consequently, there is the possibility of inappropriate recruitment of parents in less need. However, unlike a trial at a single time point, parents were recruited to a succession of groups over about 2 years.

Fourth, LAs varied in their effectiveness in organising the PEIP, including numbers of parenting groups. However, this reduced the overall impact of the PEIP compared with an analysis of the most successful LAs alone. Fifth, the measures are all parent-completed scales and parents' judgements may not reflect actual changes in their parenting styles and children's behaviours. However, previous trials of these programmes have found improvements on both the direct behavioural measures and parents' reports using standardised questionnaires such as the SDQ (e.g. [40]). Sixth, there were no follow up data available on the parents, so preventing examination of the persistence of effects. However, a long term follow up study is currently underway.

There are important implications for practice, theory and policy from the study. Regarding practice, the study shows that well designed parenting programmes, with efficacy demonstrated by time limited controlled trials, can be rolled out across a large number of community settings and the process sustained over 2 years. They can recruit substantial numbers of parents in need of parenting support and deliver significant improvements in parenting skills, parental mental well-being and child behaviour. In the present study this was achieved by central government funding of LAs within specified implementation parameters.

With respect to developing theory the finding that these three programmes, despite differences in length, style and content, were all effective raises the issue of mechanisms of change, suggesting that, in addition to a theoretically coherent content and implementation methods, relationships and style are also likely to be of importance [41]. This implies that other, well designed programmes could also be effective provided they engage parents appropriately.

Regarding policy, our evidence suggests that further roll out of these three parenting programmes to support parents is justified as a component of a policy to reduce children's behaviour difficulties. Consistency and programme fidelity in an intervention on this scale require national planning, monitoring and support of local delivery in order to limit variations in implementation and thereby enhance effectiveness. However, national and local strategy to support parents and reduce behaviour difficulties in children must be multifaceted: parenting programmes are important, but only one of a number of possible strategies [24].

## Conclusions

This study has demonstrated the individual and relative effectiveness of three parenting programmes, whose efficacy had previously been demonstrated by trials, including randomized control trials, when implemented on a large scale. The results indicate that a nationally directed but locally administered community-based implementation of well designed, evidence-based parenting programmes can improve parenting and parental mental well-being, and reduce child behaviour difficulties. Such an intervention can be developed and sustained, using any one of these three evidence-based programmes, over at least 2 years.

## Endnotes

^1^This estimate was gained by calculating the number of parents attending groups from which there were no post-course questionnaires returned at all (66 of the 267 groups containing 448 parents).

^2^The pooled (pre-course plus post-course) SD was used.

## Competing interests

The authors declare that they have no competing interests.

## Authors' contributions

GL led the research (Principal Investigator) and drafted the manuscript. SS was Co-investigator for the study, participated in its design, conducted the analyses and drafted the Results section. HD was Co-investigator, participated in its design and drafted the sections of the paper on the parenting programmes, and contributed to the overall design of the paper and the drafts. All authors read and approved the final manuscript.

## Authors' information

GL is Director of the Centre for Educational Development, Appraisal and Research (CEDAR) at the University of Warwick, England where he is also Professor of Educational Psychology and Special Needs Education. SS is Deputy Director of CEDAR and also Professor in the Institute of Education, University of Warwick. HD is Emeritus Professor of Child Health Psychology, Kings College London/Institute of Psychiatry.

## Pre-publication history

The pre-publication history for this paper can be accessed here:

http://www.biomedcentral.com/1471-2458/11/962/prepub
